# Ryegrass mottle virus complete genome determination and development of infectious cDNA by combining two methods– 3′ RACE and RNA-Seq

**DOI:** 10.1371/journal.pone.0287278

**Published:** 2023-12-05

**Authors:** Ina Balke, Ivars Silamikelis, Ilze Radovica-Spalvina, Vilija Zeltina, Gunta Resevica, Davids Fridmanis, Andris Zeltins

**Affiliations:** 1 Plant Virus Protein Research Group, Latvian Biomedical Research and Study Centre, Riga, Latvia; 2 Bioinformatics Core Facility, Latvian Biomedical Research and Study Centre, Riga, Latvia; 3 Genome Centre, Genotyping and Sequencing Unit, Latvian Biomedical Research and Study Centre, Riga, Latvia; 4 Plant Virology Group, Latvian Biomedical Research and Study Centre, Riga, Latvia; 5 “Exotic” Site Microbiome and G-Protein Coupled Receptor Functional Research Group, Latvian Biomedical Research and Study Centre, Riga, Latvia; Nuclear Science and Technology Research Institute, ISLAMIC REPUBLIC OF IRAN

## Abstract

Ryegrass mottle virus (RGMoV; genus: *Sobemovirus*) is a single-stranded positive RNA virus with a 30 nm viral particle size. It exhibits *T = 3* symmetry with 180 coat protein (CP) subunits forming a viral structure. The RGMoV genome comprises five open reading frames that encode P1, Px, a membrane-anchored 3C-like serine protease, a viral genome-linked protein, P16, an RNA-dependent RNA polymerase, and CP. The RGMoV genome size varies, ranging from 4175 nt (MW411579.1) to 4253 nt (MW411579.1) in the deposited sequences. An earlier deposited RGMoV complete genome sequence of 4212 nt length (EF091714.1) was used to develop an infectious complementary DNA (icDNA) construct for *in vitro* gRNA transcription from the *T7* promoter. However, viral infection was not induced when the transcribed gRNA was introduced into oat plants, indicating the potential absence of certain sequences in either the 5’ or 3’ untranslated regions (UTR) or both. The complete sequence of the 3’ UTR was determined through 3’ end RACE, while the 5’ UTR was identified using high-throughput sequencing (HTS)-RNA-Seq to resolve the potential absences. Only the icDNA vector containing the newly identified UTR sequences proved infectious, resulting in typical viral infection symptoms and subsequent propagation of progeny viruses, exhibiting the ability to cause repeated infections in oat plants after at least one passage. The successful generation of icDNA highlighted the synergistic potential of utilizing both methods when a single approach failed. Furthermore, this study demonstrated the reliability of HTS as a method for determining the complete genome sequence of viral genomes.

## Introduction

Ryegrass mottle virus (RGMoV) belongs to the genus *Sobemovirus* within the recently established family *Solemoviridae*, which includes the genera *Polemovirus*, *Polerovirus*, and *Enamovirus* [1]. According to the new classification of the International Committee on Taxonomy of Viruses, the genus *Sobemovirus* contains 21 assigned species and two related viruses that are unclassified and not yet assigned to a species [2]. The *Sobemovirus* genus name is derived from the reference species, ***So****uthern* ***be****an* ***mo****saic virus* [3], whereas the family name *Solemoviridae* combines the founding genera ***So****bemovirus* and *Po****lem****ovirus* [4]. *Sobemovirus* infections can lead to a wide range of symptoms in plants, including chlorotic mottling, stunting, leaf deformation, mosaic patterns, malformation, blistering, sterility, and even plant death in severe cases [5–8]. However, it is important to note that, in certain instances, *Sobemovirus* infections may proceed asymptomatically with no visible signs of disease [9]. These viruses cause diseases in economically important agricultural plants. They are responsible for the lethal yellowing disease in papaya [10], chlorotic fleck disease in ginger [11], yellow mottle disease on rice [8], and subterranean clover mottle disease of annual clover pastures [12]. RGMoV was first isolated from stunted Italian ryegrass (*Lolium multiflorum*) and cocksfoot (*Dactylis glomerate*) showing symptoms of leaf mottling and necrosis [13]. The virus represents an isometric particle with a diameter of 28 nm and contains a single-stranded positive-sense genomic RNA (gRNA) [13, 14]. The virion comprises a single-coat protein (CP) with a molecular weight (MW) of 25.6 kDa [14]. The capsid structure exhibits *T = 3* symmetry and consists of 180 CP subunits arranged in a canonical jellyroll β-sandwich fold. Compared to other sobemoviruses, the RGMoV particle is 3% smaller in diameter, and the virion cavity is 7% smaller [15]. The gRNA of RGMoV has a virus genome-linked protein (VPg) at the 5’ terminus, whereas the 3’ end lacks a poly(A) tail [14]. The gRNA encodes five open reading frames (ORFs) [16, 17]. ORF1 encodes P1, a zinc finger protein with movement functions and potential long-distance RNA silencing suppressor roles [18, 19]. ORFx-translated protein Px is responsible for establishing the infection [17]. ORF2a encodes the polyprotein P2a, which consists of three protein domains: a membrane anchor 3C-like serine protease (Pro) with a typical chymotrypsin-like protease structure [20]; a "natively unfolded" VPg protein [21] that interacts with eukaryotic translation initiation factor eIF(iso)4G [22, 23], serving for ribosome recruitment [23]; and a protein with a predicted MW of 16.8 kDa (P16) [21], with “natively unfolded protein” features [21, 24], possessing nucleic acid binding properties [21, 24] and a novel Mg^2+^-dependent ATPase activity [24]. ORF2b, located in the -1 frame relative to ORF2a, encodes the RNA-dependent RNA polymerase (RdRp) [16]. *In vitro* studies have demonstrated that the RdRp of Sesbania mosaic virus (SeMV) initiates progeny RNA synthesis in a primer-independent manner. The presence of the ACAA motif at the 3’-end of the negative-sense strand suggests that it might function as a promoter or enhancer for replication [25–27]. ORF3 of RGMoV contains a coding sequence for CP, which is translated from a subgenomic RNA (sgRNA) [15, 28]. In the case of cocksfoot mottle virus (CfMV), CP suppresses RNA silencing but is not required for CfMV cell-to-cell and systemic movement in oats, wheat, and barley [29], which differs from rice yellow mottle virus (RYMV) and southern cowpea mosaic virus (SCPMV; previously known as the cowpea strain of SBMV) [30–33]. The translation of ORFx is predicted to rely on a non-AUG initiation mechanism, whereas that of ORF2a depends on a leaky scanning mechanism [17]. Pro’s processing of polyprotein 2a occurs *in cis* at E117/A118 [20]. However, it also occurs *in trans* at E315/S316 and E393/S394, releasing mature Pro, VPg, and P16 domains [20, 34].

It is believed that each virion of sobemoviruses encapsidates one gRNA molecule and one sgRNA molecule [35]. Some sobemoviruses have been reported to encapsidate viroid-like satellite RNAs (satRNAs) dependent on a helper virus for replication [35–37]. This phenomenon has been observed in several sobemoviruses, including lucerne transient streak virus (LTSV) [38], RYMV [39], subterranean clover mottle virus (SCMoV) [40], Solanum nodiflorum mottle virus (SNMoV) [41], and velvet tobacco mottle virus (VTMoV) [41, 42]. CfMV has been shown to encapsidate the satRNA of LTSV during infection in two monocotyledonous species, *Triticum aestivum* and *D*. *glomerate* [43]. However, for CfMV, only the defective interfering RNA (diRNA) has been detected in viral particles [44].

The genome size of RGMoV deposited in GenBank and labeled as "complete genome", ranges from 4175 nt (GenBank ID MW411579.1) to 4253 nt (GenBank ID MW411579.1). The distribution of RGMoV is no longer limited to Japan. Through high throughput sequencing (HTS), RGMoV has been detected in various samples worldwide, including the United Kingdom (GenBank ID MW588198.1), USA (GenBank ID OK424596.1), Australia (GenBank ID MT129760.1), Ecuador (GenBank ID MW411579.1; [45]), and by ELISA and IEM in Germany [46]. The widespread presence of RGMoV in different regions indicates it is a plant pathogen of concern. However, there have been limited reports of RGMoV causing yield losses in fodder grasses. A previous study supporting this hypothesis reported a high incidence of RGMoV in a cultivated area of Italian ryegrass (*L*. *multiflorum* Lam.) cv. “Meroa RvP” in Saxony-Anhalt, Germany. In this area, more than 60% of the plants grown for dairy cattle feeding were infected, significantly reducing yield and forage quality [47]. This outcome is consistent with findings for *D*. *glomerate* infected with CfMV [48]. Identifying RGMoV in these diverse locations suggests the necessity for ongoing monitoring of this virus, especially considering several other sobemoviruses are of high economic importance, such as RYMV and papaya lethal yellowing virus (PLYV) [4]. For instance, RYMV is responsible for significant rice yield losses in sub-Saharan Africa, ranging from 20% to 100% [49]. This virus has also been detected in other species of the *Poaceae* family [8, 50–52] (formerly *Gramineae*), includes various grasses [53], indicating its potential to affect a wide range of plants. Similarly, PLYV poses a substantial threat to papaya plants, with infections leading to the death of up to 50% of the affected plants. Moreover, it delays fruit ripening and reduces their market value by causing the pulp to harden [54]. Plant diseases caused by viruses have a significant economic impact, with annual losses exceeding 30 billion USD [55]. They contribute to nearly 50% of emerging and re-emerging plant diseases worldwide, affecting cultivated plants and natural vegetation. These diseases threaten natural ecosystems as they can lead to alterations in the species composition of plant communities and cause genetic erosion, ultimately posing a risk of species extinction. The spread of viruses and their vectors has been exacerbated by agricultural globalization and international trade. This rapid dissemination to new geographical regions could have unexpected consequences for food production and natural ecosystems [56]. As a result, diligent monitoring and management strategies are crucial to mitigate the risks associated with the spread of these viruses and protect both agricultural systems and the environment.

The RGMoV genome was revised in previous studies, specifically concerning Pro’s amino acid (aa) sequence [14, 16]. The RGMoV coding region has been verified by resequencing [16, 57]. Various methods can be employed for non-coding regions, such as the 5’ and 3’ untranslated regions (UTRs). Classical methods, such as rapid amplification of cDNA ends (RACE), have been utilized [58], while more versatile approaches have emerged, leveraging Next-Generation Sequencing (NGS) technology, such as RACE-Seq [59]. In the case of 5’ RACE, distinguishing full-length RNA templates from degraded ones can be challenging. Additionally, difficulties may be encountered in reverse transcribing RNA sequences with high GC content because these regions can form highly stable hairpin structures from which cDNA must be derived [60]. RNA-Seq, including RACE-Seq, involves multiple steps, such as reverse transcription, PCR amplification, and NGS, which are prone to errors. For instance, PCR amplification can introduce point mutations and indels that may generate recombinant sequences or chimeras composed of two or more true template sequences. Moreover, the fraction of erroneous reads in the RNA-Seq data increases with error rate and read length. The read quality drops toward the end, leading to truncated or discarded low-quality reads. Low-quality reads must be filtered from the dataset to mitigate this issue. The quality of reads may be assessed from the base quality scores provided by the NGS platform [61]. In summary, although RACE and RACE-Seq are valuable tools for studying non-coding regions, they have inherent limitations and potential sources of error, particularly in reverse transcription, PCR amplification, and NGS steps. Researchers must be aware of these limitations and apply appropriate filtering and quality assessment methods to ensure the accuracy and reliability of their results.

Currently, RGMoV has no infectious complementary viral DNA (icDNA), hence its true genome sequence is unknown. Therefore, the concern remains regarding the genome’s 5’ and 3’ UTRs. Despite the lack of cap and poly(A) tail, sobemoviruses rely on their 5’ and 3’ UTRs to compensate for these functions [4]. A similar mechanism is observed in the tomato bushy stunt virus, where the 5’ and 3’ UTRs facilitate the translation of viral proteins through RNA-RNA interactions [62, 63]. Sobemoviruses employ a covalently bound VPg at their 5’ UTR to mimic the cap structure. VPg is linked to the 5’ phosphate group of RNA and is species-specific, with different aa residues (tyrosine for CfMV, serine for RGMoV and RYMV, and threonine for southern bean mosaic virus (SBMV)) at the N-termini of VPg [34, 64]. VPg interacts with the eukaryotic translation initiation factor, eIF(iso)4G [22, 23], to facilitate ribosome recruitment [23]. Furthermore, the 5’ UTRs of sobemoviruses may contain internal ribosomal entry sites, indicated by the presence of a polypurine tract [65], as observed in crucifer-infecting tobacco mosaic virus (TMV) [66]. Regarding the 3’ UTRs of positive-strand RNA viruses, they often form complex structures that play crucial roles in various viral processes, including translation and replication [67–69]. Computer modeling studies have identified tRNA-like structures at the 3’ UTRs of RYMV and CfMV [70, 71], but this was not observed for SBMV, SCPMV, and SeMV [72–74]. For SeMV, a specific stem-loop structure located 28–55 nt upstream from the 3’ end was identified and serves as a template recognition signal for SeMV RdRp [27]. In contrast, for CfMV, structural elements in the 3’ UTR may function as an RNA transport signal within the host [29].

Although RGMoV shares some physical and biological properties with CfMV, it is serologically distinct from CfMV and other viruses, such as cocksfoot mild mosaic virus, Cynosurus mottle virus, and Phleum mottle virus, found in European countries [14]. Despite these serological differences, RGMoV shares general properties with other sobemoviruses that infect grasses [13, 75]. The transmission vector for RGMoV remains unknown; however, similar to other sobemoviruses [4], it can be transmitted through mechanical wounding [15].

The development of the first icDNA of brome mosaic virus (BMV) by Ahlquist *et al*. in 1984 paved the way for establishing multiple reverse genomic systems based on plant viruses [76]. The construction of infectious plant virus clones has played a crucial role in advancing our understanding of these viruses at the molecular level. The generation of infectious clones represents a crucial initial step in conducting reverse genetic studies on RNA plant viruses, allowing researchers to explore the functions and sequences of viral genes [77]. These clones serve as invaluable tools for confirming Koch’s postulates, a fundamental principle in plant pathology [78], and various applications in plant science. Infectious clones are widely used to develop vectors that facilitate the expression of foreign genes in plants [79]. Moreover, they play a key role in selecting pathogen-resistant plants for breeding programs, thereby contributing to the development of disease-resistant crop varieties [80]. These clones have been instrumental in studying virus-induced RNA silencing [81]. Their implementation has opened new possibilities for functional genomics and disease control, including RNAi-enabled vaccination strategies against pathogens or invertebrate pests [82]. Furthermore, icDNA clones have enabled significant advances in characterizing viral replication processes [31, 78], and host range [83], studying viral movement within plants [84], and deciphering the functions of viral proteins [85, 86]. They have also been crucial in understanding pathogenicity mechanisms [31, 87] and the functional roles of different genomic regions within the viral genome [26]. In addition, icDNA clones have been instrumental in identifying interactions between viruses and their host plants [88] and between viruses and their insect vectors [89]. Understanding these interactions is vital for devising effective strategies for managing viral diseases and protecting agricultural systems. In the case of sobemoviruses, icDNA has been successfully generated for only five viruses: RYMV [31], CfMV [90], SCPMV [32], Sowbane mosaic virus (SoMV; previous known as Rubus chlorotic mottle virus) [91], and SeMV [26]. These icDNA constructs are valuable tools for studying the biology of viruses and investigating their interactions with host plants.

Here, we present the comprehensive genome sequence of RGMoV and describe the successful generation of icDNA for this virus. The complete RGMoV genome consists of gRNA with a length of 4287 nt. The gRNA contains a 5’ UTR of 117 nt and a 3’ UTR of 261 nt. Furthermore, we successfully reinfected plants with progeny viruses obtained from the icDNA-capped transcripts. Infection was observed after at least one passage, indicating the viability and infectivity of the icDNA-derived RGMoV. This study provides valuable insights into the biology and pathogenicity of RGMoV and opens up possibilities for further investigation of virus-host interactions and molecular mechanisms underlying RGMoV infections.

## Materials and methods

### Virus propagation and extraction

RGMoV (National Institute of Agrobiological Sciences, Japan; MAFF No. 307043) was maintained and propagated in oats (*Avena sativa* cv. Jaak) and grown in a climatic test and plant growth chamber MLR-351H (Sanyo, Osaka, Japan) under controlled conditions. The oats were grown at 19 to 23°C with a 17-hour light period.

Sap inoculation was performed on *A*. *sativa* plants using extracts obtained from the leaves of RGMoV-infected plants to subculture the virus. The infected leaves were stored at -80°C and ground in 15 mM potassium phosphate buffer (pH 7.4) using a micro pestle in a 1.5 ml tube. The mixture was gently rubbed with Carborundum (330 grit, Thermo Fisher Scientific, Waltham, MA, USA) on the upper leaf surface of 2-week-old oats, targeting the second, third, and fourth leaves.

RGMoV was purified from oat plants displaying mottling and necrotic symptoms on their leaves [13], following a previously described protocol with some modifications as described earlier [15]. Briefly, the virus was extracted from 12.82 g of oat plants and homogenized in five volumes of 0.075 M potassium phosphate buffer (pH 5.5). The sap was filtered to remove oat debris, and chloroform was added (0.4 volumes) to the filtrate. The mixture was gently agitated at room temperature (RT) for 30 min, followed by centrifugation at 5000 rpm (3214 × g) for 10 min at 4°C. The upper phase was collected and transferred to a new tube, and ammonium sulfate was added to achieve a final concentration of 25%. The solution was then mixed overnight (ON) at 4°C. The ammonium sulfate concentration was then increased to 50% (final concentration) and incubated with agitation for 3 h. Subsequently, the solution was centrifuged at 11 000 rpm (15 557 × g) for 20 min at 4°C. The resulting pellet was dissolved in 15 mM potassium phosphate buffer (pH 5.5) and dialyzed ON at 4°C in the same buffer.

The virus was further purified by sucrose density gradient centrifugation, following a protocol similar to the purification of virus-like particles from RYMV and CfMV [92]. Fractions containing the virus were dialyzed against 200 volumes of 15 mM potassium phosphate buffer (pH 5.5) and concentrated through ultracentrifugation at 72 000 rpm (280 000 × g) for 1 h at 4°C. and concentrated through ultracentrifugation at 72 000 rpm (280 000 × g) for 1 h at 4°C. The purified virus particles were solubilized in 15 mM phosphate buffer (pH 5.5), analyzed by transmission electron microscopy (TEM), and stored at 4°C for further studies.

### Genomic and total RNA extraction for 5’ and 3’ RACE, HTS and RT-PCR

To determine the 5’ and 3’ UTRs of the RGMoV genome using 5’ and 3’ RACE, the viral gRNA was isolated from 300 μg of purified viral particles. The viral particles were disassembled in a dissociation solution containing 100 mM NaCl, 10 mM TRIS-HCl (pH 8.0), 0.5% SDS, 1 mM EDTA, and 40 μg of Proteinase K (Thermo Fisher Scientific, Waltham, MA, USA). The mixture was incubated at 37°C for 1 h.

RNA was extracted by adding one volume of phenol-chloroform solution (1:1) to the disassembled viral particles, followed by centrifugation at 13 200 rpm (16 100 × g) for 10 min. RNA was precipitated by adding 1/10 volume of potassium acetate (pH 5.3) and two volumes of 96% ethanol. The mixture was incubated at -20°C for 30 min and centrifuged at 13 200 rpm (16 100 × g) for 10 min. The RNA pellet was washed with one volume of 75% ethanol and centrifuged at 13 200 rpm (16 100 × g) for 5 min. The RNA pellet was air-dried for 10 min at RT and dissolved in 30 μl of DEPC-treated water (Thermo Fisher Scientific, Waltham, MA, USA). The concentration of extracted RNA was measured using a NanoDrop-1000 spectrophotometer (Thermo Fisher Scientific, Waltham, MA, USA) and stored at -80°C until further use.

For HTS, viral gRNA was purified from a solution of 1 mg/ml viral particles. The viral particles were treated with Benzonase (25 units/μl; Novagen, Bad Soden, Germany) to remove nucleic acid from the outer space of the viral particles and the solution. The viral gRNA was then extracted from the purified viral particles using TRI REAGENT® (Sigma-Aldrich, St. Louis, MO, USA) according to the protocol. The purified RNA was dissolved in 30 μl of RNase-free water (Thermo Fisher Scientific, Waltham, MA, USA). The concentration of the purified RNA was measured using both NanoDrop-1000 and Qubit 2.0 (Thermo Fisher Scientific, Waltham, MA, USA) with a Qubit RNA high sensitivity (HS) assay kit (Thermo Fisher Scientific, Waltham, MA, USA). RNA was stored at -80°C until further use.

Total RNA for reverse transcription polymerase chain reaction (RT-PCR) was isolated from 100 mg of oat leaves using TRI REAGENT® according to the provided protocol. The purified RNA was dissolved in 30 μl of DEPC-treated water. The total RNA concentration was measured using NanoDrop-1000 and analyzed on a 0.8% native agarose gel (NAG). Total RNA was stored at -80°C.

### RGMoV gRNA 5’ and 3’ RACE verification by Sanger sequencing

The 3’ UTR of the virus was determined using the SMARTer®RACE 5’/3’ Kit (Takara Bio, Kusatsu, Japan) to determine the complete genome sequence of RGMoV and develop the icDNA construct. 2.4 μg of RGMoV gRNA was polyadenylated using Poly(A)polymerase (600 u/μl; USB, Cleveland, OH, USA). The polyadenylated RGMoV gRNA was used for first-strand cDNA synthesis according to the manufacturer’s protocol of the SMARTer®RACE 5’/3’ Kit. The 5’ and 3’ ends of the RGMoV genome were amplified separately. The 5’ end was amplified using the genome-specific (GenBank ID EF091714, version EF091714.1 (16)) primer RG-P1-R (5’ GGATCCATGATGTCTAGTCCAAGACTGCCCT 3’) and the kit-provided 10x Universal Primer Mix (10xUPS). The 3’ end was amplified using the genome-specific primer RG-seqCP-F (5’ ATCTGGGCAAGGGTCCCGCTATTCGAAG 3’) and 10xUPS. The PCR products of the 5’ and 3’ ends were extracted from the gel after analysis in 0.8% NAG using a GeneJET Gel Extraction Kit (Thermo Fisher Scientific, Waltham, MA, USA). Adenine overlaps were added to the PCR products using Taq polymerase (Thermo Fisher Scientific, Waltham, MA, USA), and the products were cloned into the linearized ddT-tailed vector pTZ-57 (Thermo Fisher Scientific, Waltham, MA, USA) using the InsTAclon PCR Cloning Kit (Thermo Fisher Scientific, Waltham, MA, USA). This step resulted in the construction of plasmids named pTZ-5’end and pTZ-3’end. The plasmid constructs pTZ-5’end and pTZ-3’end, containing the PCR fragments, were sequenced using the Sanger sequencing method. Sequencing reactions were performed using the ABI PRISM BigDye Terminator v3.1 Ready Reaction Cycle Sequencing Kit (Thermo Fisher Scientific, Waltham, MA, USA), and the sequences were determined using an ABI PRISM 3130xl sequencer (Thermo Fisher Scientific, Waltham, MA, USA). The corresponding primers M13seq-F (5’ GCC AGG GTT TTC CCA GTC ACG A 3’) and M13seq-R (5’ GAG CGG ATA ACA ATT TCA CAC AGG 3’) were used for sequencing. Sequencing reads were assembled using SeqMan software (DNASTAR, Madison, WI, USA) to generate the complete RGMoV genome sequence.

### RGMoV gRNA RNA-Seq next-generation sequencing library preparation for HTS on the ion torrent personal genome machine™ (PGM™) sequencer

Before HTS library preparation, purified gRNA from the benzonase-treated viral material was treated with DNase I (Thermo Fisher Scientific, Waltham, MA, USA) and analyzed using an Agilent 2100 bioanalyzer (Agilent Technologies, Santa Clara, CA, USA) with an Agilent HS RNA Kit (Agilent Technologies, Santa Clara, CA, USA) to assess the level of fragmentation. The gRNA was fragmented into approximately 200 nt long fragments using an S220 focused ultrasonicator (Duty Cycle 10%; intensity 5; Peak Incident Power 175 Watts; cycles per burst 200; processing time 200 s; Covaris, Woburn, MA, USA). Fragmented RNA was analyzed using an Agilent 2100 Bioanalyser with an Agilent RNA 6000 Pico kit (Agilent Technologies, Santa Clara, CA, USA) to determine the length distribution of the fragments. The concentration of the fragmented RNA was measured using a Qubit 2.0 with a Qubit RNA HS assay kit. Hybridization of the fragmented RNA (100 ng) was performed using a barcode from the Xpress RNA-Seq Barcode 1–16 Kit (Thermo Fisher Scientific, Waltham, MA, USA), Ion Adaptor Mix, and Hybridization Solution from the Ion Total RNA-Seq Kit (Thermo Fisher Scientific, Waltham, MA, USA) according to the manufacturer’s protocol. Hybridization was performed in a Veriti 96-Well Thermal Cycler (Thermo Fisher Scientific, Waltham, MA, USA) at 65°C for 10 min and 30°C for 5 min. Ligation was performed according to the Ion Total RNA-Seq Kit protocol. RT-PCR was performed at 42°C for 30 min in a Veriti 96-Well Thermal Cycler using the Ion Total RNA-Seq Kit protocol. The cDNA library was purified, and size selection was performed using Agencourt AMPure XP reagent (Thermo Fisher Scientific, Waltham, MA, USA) according to the manufacturer’s instructions. The purified cDNA library was amplified using AmpliTaq DNA Polymerase (Thermo Fisher Scientific, Waltham, MA, USA) and Ion 5’PCR and Ion 3’PCR primers on a Veriti 96-Well Thermal Cycler following the Ion Total RNA-Seq Kit protocol. The amplified DNA was purified using a PureLink PCR Micro Kit (Thermo Fisher Scientific, Waltham, MA, USA) following the manufacturer’s protocol. The purified DNA library was analyzed using an Agilent 2100 bioanalyzer with an Agilent DNA 1000 Kit (Agilent Technologies, Santa Clara, CA, USA) and a Qubit 2.0 with a Qubit 1X dsDNA HS Assay Kit (Thermo Fisher Scientific, Waltham, MA, USA) to determine the library concentration.

Emulsion PCR was prepared using the Ion OneTouch System (Thermo Fisher Scientific, Waltham, MA, USA) according to the Ion OneTouch 200 Template Kit v2 DL protocol (Thermo Fisher Scientific, Waltham, MA, USA). Sequencing was performed on an Ion 314 Chip V2 (Thermo Fisher Scientific, Waltham, MA, USA) using an Ion PGM Sequencer (Thermo Fisher Scientific, Waltham, MA, USA) and an Ion PGM Sequencing 200 Kit (Thermo Fisher Scientific, Waltham, MA, USA) following the standard protocol.

### HTS data analysis

After the gRNA RNA-Seq library preparation, the sequencing reads underwent additional processing and analysis steps. Adapter, barcode, and quality trimming were performed with cutadapt version 4.2 [93] and fastp version 0.23.2 [94]. Reads with lengths of at least 50 bp were retained for further analysis. The trimmed reads were used for contig assembly using SPAdes genome assembler v3.15.5 [95]. The assembly was performed in "rnasviralSPAdes" mode with the "iontorrent" flag set on, indicating the specific sequencing platform used. Bowtie2 (version 2.3.5.1) [96] was used for read mapping against the assembled contigs and reference sequences. In order to identify reads related to additional sequences in the 5’ UTR region, manual sequence alignments were performed using SeqMan software. The resulting data were visualized using Matplotlib v3.6.3 [97].

### RGMoV icDNA vector construction under *T7* polymerase promoter

The cDNA construct containing the RGMoV genome sequence from GenBank under accession number EF091714 has been reported previously [16]. The 3’ UTR with an additional 52 nt sequence obtained by 3’ RACE was amplified from the pTZ-3’end plasmid. iProof High-Fidelity DNA Polymerase (500 U; Bio-Rad, Hercules, CA, USA) was used for the amplification, along with primers RG-seqCP-F and RG-3pN-Bst-R (5’ CAGTATACTACAACCCCTAGCGTGGAATGATCCTACCCTAGGCA 3’). The PCR reaction was performed on a Veriti 96-Well Thermal Cycler at an annealing temperature of 55°C. The resulting PCR product was extracted from the gel using a GeneJET Gel Extraction Kit, and adenine overlaps were added using Taq polymerase. The PCR product was then cloned into the linearized vector pTZ-57 using the InsTAclon PCR Cloning Kit, generating the plasmid construct pTZ-3’end-PCR. Clones containing the PCR fragments were verified using Sanger sequencing. The pTZ-3’end-PCR clone and a plasmid containing RGMoV gRNA were digested with BamHI restriction enzyme (Thermo Fisher Scientific, Waltham, MA, USA). DNA fragments were extracted from the gel using the GeneJET Gel Extraction Kit and ligated to generate a cDNA construct named pJET-RGMoV-cDNAp-3end. The constructed plasmid was analyzed by restriction digestion and verified by Sanger sequencing. HTS identified two variants of the 5’ end sequence. The first construct was developed using a 36 nt fragment (5’UTR-new). Two-step PCR was performed to amplify the 5UTR-new fragment. The first PCR was carried out using primers RG-5UTR-new-F (containing *T7* promoter sequence; 5’ACCATCGCGATAATACGACTCACTATAGGGACCTCTCTATGGGCAGTCTCCTCTCTATGGCAGTCGACAAA 3’) and RG-5UTR-new-R (5’TCACCGGATAACGGGTTCAATAGAGTTAATTTAATAACTCTATTTGTCGACTGCCATAGAGAGGAGACTG 3’) and Pfu polymerase (Thermo Fisher Scientific, Waltham, MA, USA). The PCR products were extracted from the gel. The second PCR product was amplified from the purified PCR product and a plasmid containing the RGMoV first gRNA fragment, pTZ-1fr [16]. After 5 cycles of amplification with an annealing temperature of 55°C primers RG-5UTR-new-F and RG2-P1-HindIII-R (5′ AAGCTTATGATGTCTAGTCCAAGACTGCCCTCGA 3′) were added followed by 25 cycle amplification at annealing temperature at 55°C. PCR fragment was extracted from the gel using the GeneJET Gel Extraction Kit. Adenine overlaps were added and cloned into the linearized vector pTZ-57 using the InsTAclon PCR Cloning Kit. The resulting plasmid construct was named pTZ-5UTR-new and verified using Sanger sequencing. The pTZ-RGMoV-5UTR-new and pJET-RGMoV-cDNA-3end constructs were cleaved by Eco91I (BstEII; Thermo Fisher Scientific, Waltham, MA, USA) and PstI (Thermo Fisher Scientific, Waltham, MA, USA) restriction enzymes. DNA fragments were extracted from the gel using the GeneJET Gel Extraction Kit and ligated to obtain the construct pTZ-T7-RGMoV-cDNA-3end-5UTR-new-PGM. A 5’ end construct with a 19 nt addition (5’UTR-new-short) was created using the pTZ-RGMoV-5UTR-new plasmid as a template. PCR amplification was performed with Pfu polymerase using primers RG-P1-R and RG-5UTR-new-short-F (containing the *T7* promoter sequence; 5’ ACCATCGCGATAATACGACTCACTATAGGGACCTCTCTATGGGCAGT 3’). The PCR products were extracted from the gel; adenine overlaps were added and cloned into the pTZ-57 cloning vector. The resulting plasmid, pTZ-RG-5UTR-new-short, was verified by Sanger sequencing. pTZ-RG-5UTR-new-short and pTZ-T7-RGMoV-cDNA-3end-5UTR-new-PGM were subjected to digestion with Mph1103I (NsiI; Thermo Fisher Scientific, Waltham, MA, USA) and Eco91I restriction enzymes. The resulting RG-5UTR-new-short fragment was ligated into the pTZ-T7-RGMoV-cDNA-3end-5UTR-new-PGM plasmid. This ligation process generated the final icDNA construct named pTZ-T7-RGMoV-cDNA-3end-5UTR-new-short-PGM.

### RGMoV gRNA *in vitro* synthesis

**Fig 1:**
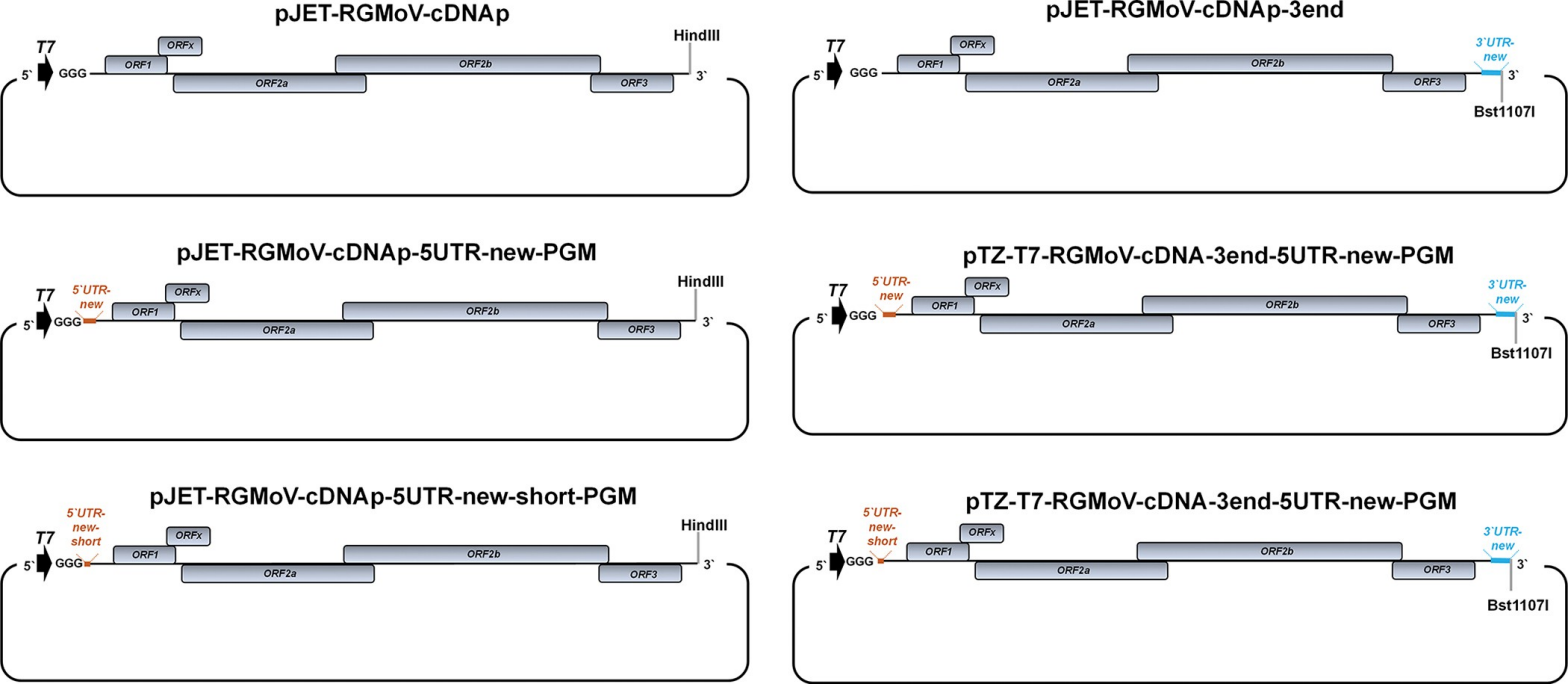
Schematic overview of created cDNA construct used for RNA transcription and oat plant inoculation. *T7* –*T7* polymerase promoter; blue marking represents the 3’ UTR novel sequence identified by 3’ RACE; orange marking represents the 5’ UTRs novel sequence identified by HTS RNA-Seq.

Before RNA synthesis, all icDNA constructs (Fig 1), including pJET-RGMoV-cDNAp, pJET-RGMoV-cDNAp-5UTR-new-PGM, pJET-RGMoV-cDNAp-5UTR-new-short-PGM, pJET-RGMoV-cDNAp-3end, pTZ-T7-RGMoV-cDNA-3end-5UTR-new-short-PGM, and pTZ-T7-RGMoV-cDNA-3end-5UTR-new-PGM, were subjected to linearization by restriction digestion. The plasmids (10 μg) pJET-RGMoV-cDNAp, pJET-RGMoV-cDNAp-5UTR-new-short-PGM, and pJET-RGMoV-cDNAp-5UTR-new-PGM were digested with HindIII (Thermo Fisher Scientific, Waltham, MA, USA), whereas pJET-RGMoV-cDNAp-3end, pTZ-T7-RGMoV-cDNA-3end-5UTR-new-short-PGM, and pTZ-T7-RGMoV-cDNA-3end-5UTR-new-PGM were digested with Bst1107I (Thermo Fisher Scientific, Waltham, MA, USA). To verify the success of the linearization process, purified DNA was analyzed using NAG electrophoresis (NAGE) on a 0.8% agarose gel stained with ethidium bromide (Thermo Fisher Scientific, Waltham, MA, USA). The concentration of the linearized DNA was estimated using a NanoDrop-1000 spectrophotometer.

Capped gRNA was synthesized using either the RiBoMax Large Scale RNA Production Systems (Promega, Madison, Wis, USA) carried out at 30°C according to the protocol or the TranscriptAid T7 High Yield Transcription Kit (Thermo Fisher Scientific, Waltham, MA, USA) following the respective protocol provided by the manufacturers for *in vitro* RNA transcripts with or without a cap. The Cap Analog used for transcripts with a cap was the Ribo m7G Cap Analog (40 mM; A254 units, Promega, Madison, Wis, USA, P171B). After synthesis, RNA was purified using the RNeasy Plant Mini Kit (Qiagen, Hilden, Germany) according to the manufacturer’s protocol. It was analyzed using NAGE on a 1% agarose gel stained with ethidium bromide to verify the integrity of the synthesized RNA. The concentration of purified RNA was estimated using a NanoDrop-1000 spectrophotometer.

### RGMoV gRNA transcript coating on gold microcarriers for Helios Gene Gun

One 60 cm Gold-Coat tubing and 25 mg of Au microcarriers with 1 μm Ø were used from the Helios Gene Gun Optimization Kit (Bio-Rad, Hercules, CA, USA) to prepare the cartridges for gene gun delivery. 50 μg of the synthesized RNA transcript per 25 mg of Au microcarriers were used to reach 0.5 mg a microcarrier loading quantity and 1 μg of the RNA loading ratio. A Tubing Prep Station (Bio-Rad, Hercules, CA, USA) was used to coat the gold-coated tubing with Au-RNA microcarriers. All procedures were performed according to the Helios Gene Gun System instruction manual.

### Oat inoculation with gRNA transcripts

Oat plants were inoculated with the gRNA transcript in the first leaf at the second-leaf stage using two different methods: mechanical rubbing or bombardment with Au microcarriers coated with RNA. Ten control plants were mock-inoculated with DEPC-treated water.

Each RGMoV gRNA transcript variant group contained ten oat plants. For oat plants inoculated with the gRNA transcript by mechanical rubbing, 2.5 μg of the gRNA transcript, capped or cap free, was mechanically rubbed onto the first leaf of oat plants using Carborundum as an abrasive agent. The Au-RNA microcarriers were bombarded onto the oat bombardment group plants using a Helios Gene Gun (Bio-Rad, Hercules, CA, USA) at a pressure setting of Hpsi = 200. The first inoculation was performed when the oat plants reached the second-leaf stage. After one week, a second inoculation was conducted on the first leaf. Another week later, a third inoculation was performed at the third leaf stage on the second leaf. The oat plants were grown under the same conditions as those used for virus propagation. The plants were allowed to grow for four weeks after the third inoculation. Samples for sodium dodecyl sulfate–polyacrylamide gel electrophoresis (SDS-PAGE), Western blotting (WB), and total RNA extraction for RT-PCR were collected from the fourth (systemic) leaf of oat plants. RT-PCR was performed using the Verso 1-Step RT-PCR Hot-Start kit (Thermo Fisher Scientific, Waltham, MA, USA). The primers used for RT-PCR were RGCP-NdeI-F (5’ ATGGCAAGGAAGAAGGGCAAATCGGCCA 3’) and RGCP-Pich-EcoRI-R (5’ GGAATTCTCACTGGTTGATTGTGACATCAACCGGA 3’). The RT-PCR protocol provided by the manufacturer was used for the amplification.

### Transmission electron microscopy (TEM)

Purified virus samples were adsorbed onto carbon formvar-coated grids (300 mesh Copper/Palladium 3.05 mm; Laborimpex, Forest, Belgium) or carbon formvar-coated grids (hexagonal 200 mesh nickel 3.05 mm; Laborimpex, Forest, Belgium) and negatively stained with a 0.5% uranyl acetate aqueous solution. Stained grids were examined using a JEM-1230 TEM (JEOL, Tokyo, Japan) at an accelerating voltage of 100 kV, as described previously [98].

### Oat leaf extract analysis by SDS-PAGE and Western blot

100 mg of oat leaf was ground in a 1.5 ml tube with 200 μl 2xLaemmli sample buffer (100 mM Tris-HCI (pH 7.0), 2% SDS, 50% glycerol, 0.005% Bromophenol Blue, and 2% mercaptoethanol) using a micro pestle (Kontes, Vineland, New Jersey, USA). Before loading onto a 12.5% SDS-PAG [92], the proteins were incubated at 95°C for 10 min. After SDS-PAG were stained with R250 (10% (v/v) ethanol, 10% (v/v) glacial acid, and 0.1% (w/v) R250) or G250 (20% (v/v) ethanol, 2% (w/v) trichloroacetic acid, and 0.05% (w/v) G-250).

WB was performed on a semi-dry WB device and transferred to an Amersham Protran 0.45 mm nitrocellulose blotting membrane (GE Healthcare, Chicago, IL, USA) at 52 mA for 45 min. The blocking and subsequent blotting steps were performed in a 1% alkali-soluble casein solution (MerckMillipore, Darmstadt, Germany). The membrane was blocked for 1 h at RT on a Mixer 820 (Boule Medical, Stockholm, Sweden) at 60 Hz 18 rpm/8 rpm. After blocking, the membrane was blotted ON at 4°C with polyclonal anti-RGMoV (dilution 1:1000; produced in-house against wild-type (WT) RGMoV targeting CP). The membrane was washed with 15 ml of TBS buffer (150 mM NaCl; 10 mM Tris, pH 7.5) for 15 min on Mixer 820 at 60 Hz at 18 rpm/8 rpm at RT. Incubation with horseradish peroxidase-conjugated anti-rabbit IgG (1:1000; Sigma-Aldrich, St. Louis, MO, USA) was performed at RT for 3 h. The membrane was washed with TBS for 15 min on a Mixer 820 at 60 Hz 18 rpm/8 rpm at RT. The WB was visualized with TBS buffer supplemented with peroxidase substrate (0.002% (w/v) o-dianisidine and 0.03% (v/v) hydrogen peroxide) and incubated in the dark at 37°C for 30 min. The reaction was terminated by washing the membrane with dH_2_O.

## Results and discussion

**Fig 2:**
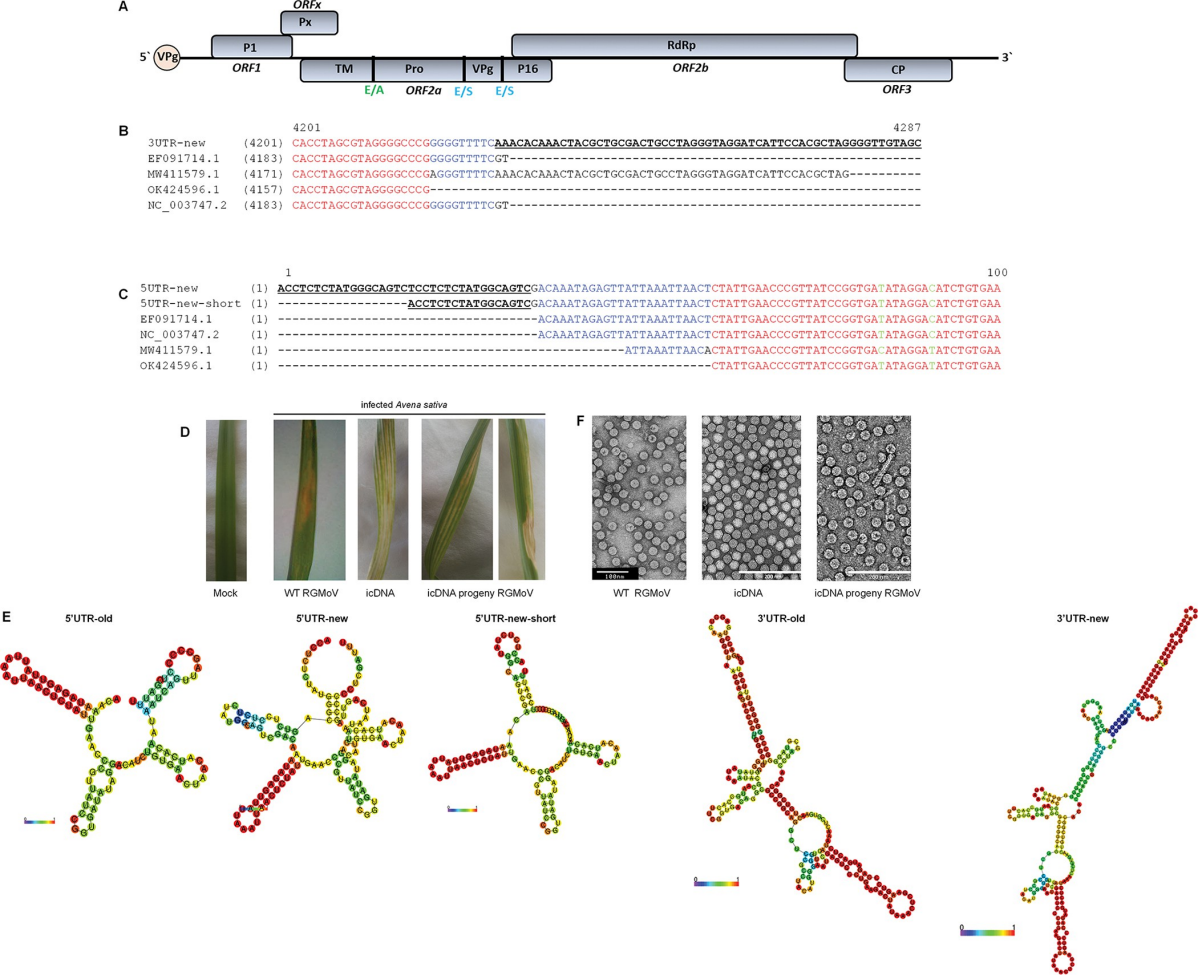
RGMoV icDNA 5’ and 3’ UTR sequence and progeny virus detection. A–schematic overview of RGMoV genome; B–schematic sequences of newly identified RGMoV icDNA 3’ UTRs in comparison of in GenBank deposited RGMoV complete genome sequences; C–schematic sequences of newly identified RGMoV icDNA 5’ UTR in comparison of in GenBank deposited RGMoV complete genome sequences; D–comparison of Mock and infected with WT RGMoV, icDNA capped RNA transcript, and reinfected with icDNA derived progeny virus *A*. *sativa* plant leaves bearing RGMoV classical infection pattern; E–comparison of the minimum free energy RNA structures of old and novel identified 5’ and 3’ UTRs created by RNAfold WebServer [99]; F–transmission electron microscopy analysis of purified virus particles from WT RGMoV, icDNA capped RNA transcript, and reinfected with icDNA derived progeny virus; bar represents 200 nm scale; red and blue marked nucleotides (nt) represents sequences deposited in GenBank and are conserved between isolates; underlined black bold marked nt represent the sequences identified in this research; green marked nt represents single nt changes in deposited sequences.

The development and successful testing of icDNAs derived from viral gRNA can be considered a critical and conclusive step in confirming the completeness and authenticity of the virus genome. Prior to this, multiple techniques, such as Sanger sequencing and 5’ and 3’ RACE, were employed to determine the viral genome’s nt sequence. However, these methods alone may not be sufficient to establish viruses’ functional and biological properties. Infectious clones constructed using viral gRNA serve as a crucial tool to validate the accuracy of the sequenced viral genome and confirm that the obtained sequence represents a functional virus that can effectively infect host cells. The ability of icDNA to replicate and produce infectious viral particles provides strong evidence that viral genome sequences are accurate. The icDNA construct derived from the RGMoV gRNA sequence, specifically the pJET-RGMoV-cDNAp construct [16], failed to induce virus infection in oat plants (S1 Fig), indicating the importance of correct 5’ and 3’ ends in the construct. In an attempt to determine the UTRs of the viral genome, both 5’ and 3’ RACE methods were employed. However, only the 3’ UTR was successfully identified, revealing that it was 52 nt longer than previously published sequences [14, 16, 57] and 13 nt longer than the recently published complete genome of RGMoV (GenBank ID MW411579.1) (Fig 2B). Despite efforts to develop the icDNA clone pJET-RGMoV-cDNAp-3end, which included the extended 3’ UTR, this construct also failed to induce local or systemic infection in oat plants (S1 Fig). Subsequently, alternative methods were explored to determine the 5’ UTR. Multiple attempts using RACE, various RT-PCR conditions, and primers were unsuccessful. In addition, RNA circularization using T4 RNA ligase did not yield the desired results. Ultimately, the identification of additional sequences in the 5’ UTR end was achieved by analyzing RGMoV gRNA RNA-Seq HTS data. The identification highlights the importance of using multiple approaches to determine the true UTR sequence.

Through sequence alignment of RNA-Seq HTS data, two sequences were identified for the 5’ UTR of the RGMoV genome: one sequence, referred to as 5UTR-new, was 36 nt longer than the reference sequence (GenBank ID EF091714.1; [16]; Fig 2C), and the other sequence, called 5UTR-new-short, was 19 nt longer (Fig 2C). Only one read corresponding to 5UTR-new (S2A Fig) and two reads corresponding to 5UTR-new-short (S2B Fig) were identified, with an average coverage of 155.9 reads (S2A Fig) and 176.8 reads (S2B Fig), respectively, for the respective UTRs. Attempts were made to construct icDNA clones using these UTR sequences in combination with the 3’ UTR from the reference genome. Specifically, the clones pJET-RGMoV-cDNAp-5UTR-new-PGM and pJET-RGMoV-cDNAp-5UTR-new-short-PGM contained the 5UTR-new and 5UTR-new-short sequences, respectively, along with the reference 3’ UTR. However, both constructs failed to induce viral infection (S1 Fig).

Interestingly, the icDNA clone pTZ-T7-RGMoV-cDNA-3end-5UTR-new-short-PGM, which contained the 5UTR-new-short sequence and a new 3’ UTR, was able to induce systemic viral infection with corresponding infection symptoms, such as mottling and necrotic symptoms on leaves (Fig 2D). This outcome is consistent with previous studies [13]. Regarding the 5UTR-new sequence, it is mentioned that it could potentially be an artifact of HTS library preparation methods or an error during the sequencing process. This finding suggests the need for further investigation and validation to confirm this sequence’s authenticity and biological relevance.

These findings highlight the complexities and challenges in characterizing UTRs and their impact on viral infectivity, emphasizing the importance of careful experimental design and validation in viral research.

The analysis of possible RNA structures using the RNAfold WebServer [99] has provided valuable insights into the structural features of the 5’ and 3’ UTRs of the RGMoV icDNA. The predicted structure of the 5’ UTR revealed a small loop with a high base-pairing probability (Fig 2E), which may explain the difficulties encountered during 5’ RACE and the low read coverage observed for HTS RNA-Seq (S2A and S2B Fig). This small loop structure, in conjunction with the VPg protein, could potentially hinder the efficiency of the 5’ RACE method. On the other hand, the predicted structure of the 3’ UTR of the icDNA exhibited a stem-loop structure with a high base-pairing probability (Fig 2E). This structural feature was absent in all other non-infectious cDNA clones. Similar stem-loop structures have been reported for other sobemoviruses [4, 35]. This finding highlights the significance of stem-loop structures in facilitating proper viral genome transcription.

The presence of these distinct structural elements in the UTRs of RGMoV icDNA suggests their importance in the viral replication cycle and supports their role in facilitating viral genome transcription. Understanding the structural features of the UTRs can provide valuable insights into the mechanisms underlying viral replication and gene expression.

**Fig 3:**
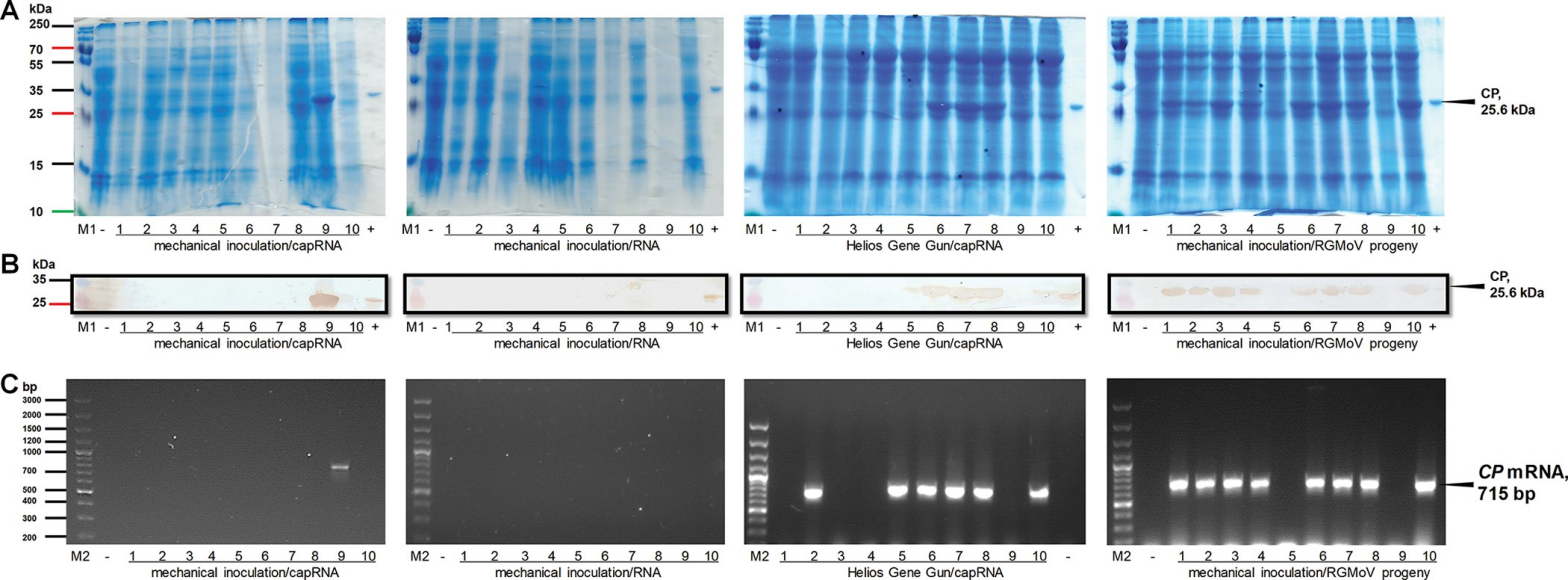
RGMoV icDNA *in vitro* RNA transcript infectivity test in *Avena sativa*. A–*A*. *sativa* leaf sample analysis in SDS-PAGE stained with G-250; B–*A*. *sativa* leaf sample analysis by WB (primary antibodies: in-house produced anti-rabbit WT RGMoV polyclonal antibodies targeting CP in dilution 1:1000; secondary antibodies: horseradish peroxidase-conjugated anti-rabbit IgG in dilution 1:1000; Sigma-Aldrich, St. Louis, MO, USA); C–RT-PCR targeting RGMoV *CP* mRNA; M1 –protein marker (Page Ruler Plus, Thermo Fisher Scientific, Waltham, MA, USA); M2 –DNA marker (GeneRuler 100 bp Plus DNA Ladder, Thermo Fisher Scientific, Waltham, MA, USA); “-”–negative control mock-inoculated *A*. *sativa*; 1–10 –with icDNA transcribed RNA or icDNA progeny virus inoculated *A*. *sativa* replicates; “+”–positive control purified WT RGMoV.

In this study, leaf samples were collected and used for the detection of the CP signal by SDS-PAGE (Fig 3A), WB (Fig 3B) with polyclonal antibodies raised from the WT virus targeting CP, and RT-PCR of the *CP* gene (Fig 3C). The results showed that when the gRNA transcript was inoculated by mechanical rubbing, only one out of 10 oat plants was infected (Fig 3). However, when the gRNA transcript was inoculated using the Helios Gene Gun (HGG) method, six out of 10 oat plants were infected (Fig 3). This finding demonstrated that the HGG method was more appropriate and effective for *in vitro*-produced viral gRNA inoculation because Au-RNA microcarriers were delivered directly into cells.

No viral infection was detected in uncapped RNA transcripts (Fig 3), suggesting that a cap structure in the RNA transcript is important for successful viral infection. Another study, which first demonstrated the development of icDNA for RYMV, showed that the capped RNA *in vitro* transcript from icDNA induced a disease phenotype identical to that produced by inoculated WT viral RNA. However, the RNA transcript of RYMV icDNA was less effective. RYMV icDNA RNA transcripts without caps showed no disease symptoms four weeks after inoculation [31]. This result highlights the importance of VPg covalent binding to RNA in the viral genome transcription process. VPg, found in other plant viruses of the family *Potyviridae*, is a multifunctional protein involved in viral replication and movement [100]. It interacts with the host cap-binding eIF4E or its isoform (eIF(iso)4E) to initiate viral genome translation [101]. For sobemoviruses, the interaction between RYMV VPg and the central domain of rice eIF(iso)4G1 has been experimentally determined [22, 23]. These findings provide insights into the importance of viral RNA structure, including the cap structure and VPg, in viral infection and replication processes.

TEM analysis of the derived icDNA RNA transcript and its progeny virus-purified viral fraction revealed typical icosahedral viral particles with an overall diameter of 30 nm, resembling WT RGMoV (Fig 3E) [15]. This result confirmed the successful production of intact viral particles. In a study of SeMV, deletion mutants of the 5’ and 3’ UTRs showed that the nt at the fourth position from the 3’ end or nt at the fifth position from the 5’ end is not crucial for infectivity. However, these mutations resulted in poor CP accumulation and unsuccessful purification of the progeny virus [26]. In the case of RGMoV, CP accumulation was high, and progeny virus purification was successful (Fig 2F), indicating that the sequenced and cloned icDNA corresponded to the complete viral genome.

The first nt of RGMoV 5’ gRNA is A, similar to RYMV [64] and SBMV [34], which agrees with the VPg-binding position identified at the first serine residue [34]. In the case of RYMV, poorer infectivity was attributed to the presence of an extra 5’ non-viral G residues, which were added to enhance *in vitro* transcription levels [31]. In RGMoV, three additional G residues were added as part of the sequences from positions +4 to +8 downstream of the transcription start site, which were found to affect *T7* promoter activity [102]. Similar situations have been observed for viruses with deviations from the original sequences, such as lacking a native 5’ G residue [103]. In the case of SoMV, the transcribed RNA carried a non-templated G at the 5’ terminus and three non-viral C residues at the 3’ terminus. This RNA was infectious in *Chenopodium quinoa’s* test plant but not in *Nicotiana benthamiana* [91]. These findings highlight the importance of specific nt sequences and structures in viral infectivity, CP accumulation, and successful replication. The proper alignment and preservation of the viral genome sequence and structure are crucial for maintaining viral functionality.

The 5’ and 3’ RACE method has been widely used to determine the transcription start point(s) and poly(A) tail sites of mRNA transcripts or viral RNA and is considered the main strategy for UTR determination [59]. However, there are cases where traditional RACE methods or alternative approaches fail to identify UTRs accurately. In this study, we found that RNA-Seq could be successfully applied to overcome the limitations of traditional methods for UTR determination in the development of icDNA. RNA-Seq is a sequencing-based approach that provides comprehensive information about the transcriptome, including identifying UTRs. Oxford Nanopore Technologies described a sequencing approach called 5’ RACE-Seq, which combines elements of RACE and RNA-Seq to determine the 5’ ends of transcripts [59]. The RACE and RNA-Seq methods use adapters to capture and amplify the ends of RNA molecules, allowing for the determination of UTR sequences. The results obtained from these methods have a similar probability of being true, and the accuracy of the results depends on statistical analysis and validation. In our study, the accuracy of UTR sequences was validated through the propagation of icDNA and testing its infectivity in progeny virus assays. By utilizing RNA-Seq or alternative sequencing approaches, researchers can overcome the limitations of traditional UTR determination methods and obtain comprehensive and reliable information about the transcriptome, including its sequences.

In our study, we tested the infectivity of the progeny virus by sap inoculation from icDNA-derived RNA-infected plants. SDS-PAGE (Fig 3A), WB (Fig 3B), and RT-PCR (Fig 3C) analyses of inoculated oat plants with icDNA-derived RNA progeny viruses showed that eight out of 10 infected plants were RGMoV-positive (Fig 3). The observed 80% infectivity rate could be attributed to genetic variability among the oat seedlings used in the experiment.

Further analysis should be conducted to better understand the observed infectivity and identify potential differences that may contribute to the infectivity rate. Transcriptome RNA-Seq analysis or analysis of eIF sequences from infected and non-infected plants may be performed. This analysis could help identify any genetic or transcriptional differences between plants associated with progeny virus infectivity. It also sheds light on the impact of RGMoV on the transcriptional patterns of host genes, providing valuable insights into plant responses to viral infection.

This study opens new avenues for investigating various aspects of RGMoV, including its replication, RNA packaging, interactions with the host, genome modifications, transmission, symptomatology, and host range. The use of icDNA and the analysis of progeny viruses provides researchers with a powerful tool for studying genomic and viral life cycle dynamics and their impact on host plants.

Rutgers *et al*. (1980) demonstrated that purified viral particles of SBMV contained minor amounts of sgRNA capable of inducing the synthesis of CP in wheat embryo extracts and reticulocyte lysates. Similar findings were reported for tymoviruses and TMV, where minor amounts of sgRNA were encapsidated, while bromoviruses encapsidated sgRNA in significant quantities [104–106]. However, Cho and Dreher showed that the sgRNA of the tymovirus turnip yellow mosaic virus was not encapsidated into viral particles [107]. In the case of sobemoviruses, such as LTSV, RYMV, SCMoV, SNMoV, and VTMoV, satRNAs can be encapsidated along with the gRNA [38–42]. CfMV was found to encapsidate satRNA of LTSV in a host-dependent manner [35, 43], and the encapsulation of diRNA was also observed [44]. Analyzing RNA-Seq HTS data from the Ion Torrent PGM to identify encapsulated sgRNA is challenging because the sgRNA sequence is embedded within the gRNA. In the case of RGMoV, the 5’ end of the sgRNA could potentially start at position 2844, where a complementary sequence of five nt from the 5’ UTR (ACAAA) was located. A similar sequence was identified in CfMV, suggesting a hypothetical start site of sgRNA [108]. The higher coverage of certain regions in the CP (S2C Fig) coding sequence compared to other regions in the gRNA could indirectly indicate the presence of sgRNA in the analyzed sample, as the higher availability of the *CP* matrix sequence suggests the possible presence of sgRNA. Oxford Nanopore technology could be a great solution for the true sequence determination of RGMoV sgRNA. Differences in coverage could be attributed to factors such as more efficient amplification of certain regions or protection of those regions by CP from RNases during viral storage. These regions may be more abundant and potentially interact with the inner cavity of the virion, potentially serving as the origin of assembly (OAS) during virus assembly. As observed, after virus treatment with RNase A, the extracted RNA concentration was too low for HTS. OAS and packaging signals have been identified in several plant and animal viruses and phages [109–114]. However, identifying OAS in RGMoV gRNA in previous studies was unsuccessful. Thus, siRNA-Seq may be used for the identification of OAS.

TEM analysis of WT RGMoV also revealed the presence of particles with symmetry resembling *T = 1* (S1 Raw images), suggesting the possibility of encapsulating sgRNA within them. This phenomenon was also observed in BMV, where the encapsulated RNA controlled the capsid structure. gRNA was packaged into 180 subunit particles, whereas mRNA containing only BMV *CP* was packaged into 120 subunit particles, demonstrating that RNA features can influence ribonucleoprotein complexes and alternative structural pathways [115]. This finding could be explained by SBMV sgRNA encapsidate rate [28]. The structural differences between the RGMoV CP and other sobemoviruses with known CP 3D structures, such as CfMV [116], RYMV [117, 118], SeMV [119], SBMV [120], and SCPMV [118, 121], may also support the hypothesis of sgRNA encapsidation. Notably, the absence of an FG loop in the RGMoV CP decreases the size of the virion cavity by 7% [15].

In conclusion, the encapsidation of sgRNA in viral particles is a complex phenomenon observed in various viruses. Further investigations, including the analysis of RNA-Seq data, siRNA-Seq, Oxford Nanopore, and structural studies, can provide valuable insights into the presence and role of sgRNA in RGMoV and shed light on its impact on viral replication, assembly, and other aspects of the viral life cycle.

Our study is the first to report an infectious and mechanically transmittable full-length cDNA clone of RGMoV. Successful determination of the full RGMoV genome sequence provides researchers with an important tool for studying sobemoviruses regarding plant reverse genomics and viral life cycle investigations.

This newly developed methodological approach, which combines RNA-Seq and short-read sequencing platforms such as the Ion Torrent PGM, demonstrates the compatibility and efficacy of RNA-Seq in generating comprehensive sequence information. This approach enhances our understanding of various aspects of sobemovirus biology, including replication, RNA packaging, host-virus interactions, genome modifications, transmission mechanisms, symptomatology, and host range.

Overall, the availability of the full-length cDNA clone and the complete genome sequence of RGMoV (updated GenBank ID EF091714 to version EF091714.2) opens new avenues for further research and provides a valuable resource for the scientific community studying sobemoviruses. Furthermore, cloned viral genome copies have potential applications beyond fundamental research. It may be utilized as a valuable heterologous protein expression vector for biotechnological purposes, potentially enhancing the development of innovative strategies for protein expression and other related biotechnological endeavors in plant farming.

## Supporting information

S1 Fig Oat leaf sample analysis after inoculation with RGMoV *in vitro* synthesized gRNA. A–oat leaf sample analysis in SDS-PAGE stained with R-250 or G-250; B–oat leaf sample analysis by Western blot with primary anti-rabbit polyclonal antibodies raised against WT virus (produced in-house) in dilution ratio 1:1000 and secondary antibodies horseradish peroxidase-conjugated anti-rabbit IgG (1:1000; Sigma-Aldrich, St. Louis, MO, USA); M–protein marker (Page Ruler Plus, Thermo Fisher Scientific, Waltham, MA, USA); “-”–mock plants; 1–10 –inoculated oat plants with corresponding cDNA RNA transcript; “+”–purified WT RGMoV as positive control.
(TIF)

S2 Fig Schematic of RNA-Seq high-throughput sequencing read alignment. A–read alignment for 5’UTR-new, reads with lengths of at least 50 bp; B–read alignment for 5’UTR-new-short, reads with lengths of at least 50 bp; C–read alignment of the RGMoV genome, reads with lengths of at least 50 bp.
(TIF)

S1 Raw images WT RGMoV analysis by TEM. Red arrows indicate possible RGMoV *T = 1* particles; the white bar represents the 500 nm scale.
(PDF)
