# Supplementary figures and images for: Ryegrass mottle virus complete genome determination and development of infectious cDNA by combining two methods– 3′ RACE and RNA-Seq
Source: PLoS One. 2023 Dec 5;18(12):e0287278. doi: 10.1371/journal.pone.0287278 (PMC10697606; doi:10.1371/journal.pone.0287278)

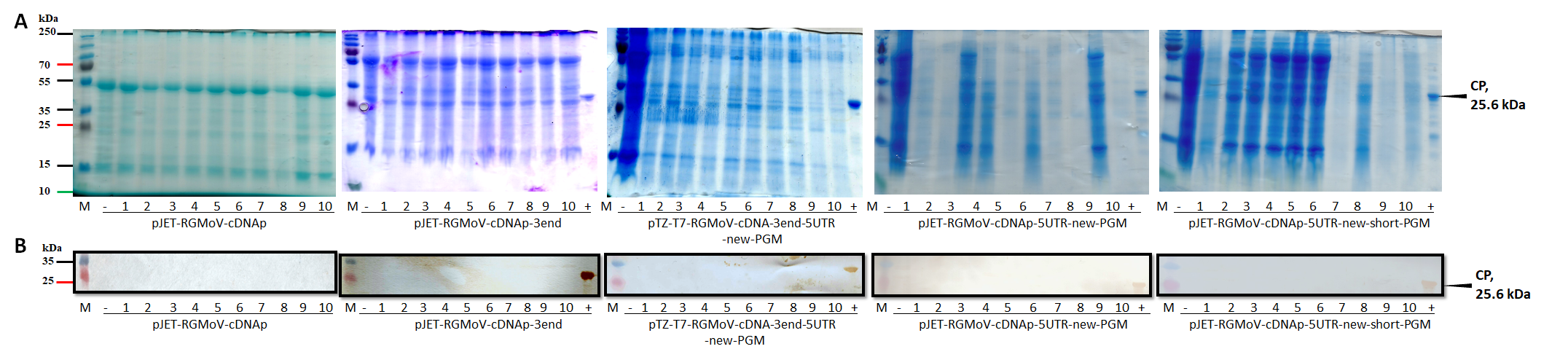

Supplement: S1 Fig — A–oat leaf sample analysis in SDS-PAGE stained with R-250 or G-250; B–oat leaf sample analysis by Western blot with primary anti-rabbit polyclonal antibodies raised against WT virus (produced in-house) in dilution ratio 1:1000 and secondary antibodies horseradish peroxidase-conjugated anti-rabbit IgG (1:1000; Sigma-Aldrich, St. Louis, MO, USA); M–protein marker (Page Ruler Plus, Thermo Fisher Scientific, Waltham, MA, USA); “-”–mock plants; 1–10 –inoculated oat plants with corresponding cDNA RNA transcript; “+”–purified WT RGMoV as positive control. (TIF) [file pone.0287278.s001.tif]

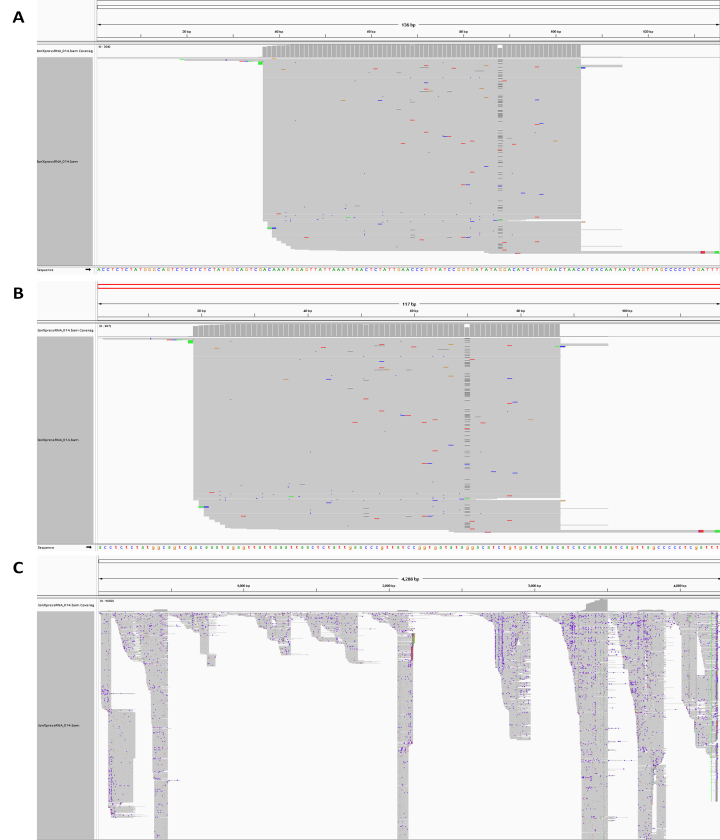

Supplement: S2 Fig — A–read alignment for 5’UTR-new, reads with lengths of at least 50 bp; B–read alignment for 5’UTR-new-short, reads with lengths of at least 50 bp; C–read alignment of the RGMoV genome, reads with lengths of at least 50 bp. (TIF) [file pone.0287278.s002.tif]
