# Supplementary material for: Ryegrass mottle virus complete genome determination and development of infectious cDNA by combining two methods– 3′ RACE and RNA-Seq
Source: PLoS One. 2023 Dec 5;18(12):e0287278. doi: 10.1371/journal.pone.0287278 (PMC10697606; doi:10.1371/journal.pone.0287278)

DNA and protein ladders used in gels and blots

GeneRuler 100 bp Plus DNA Ladder;
Catalog number: SM0321

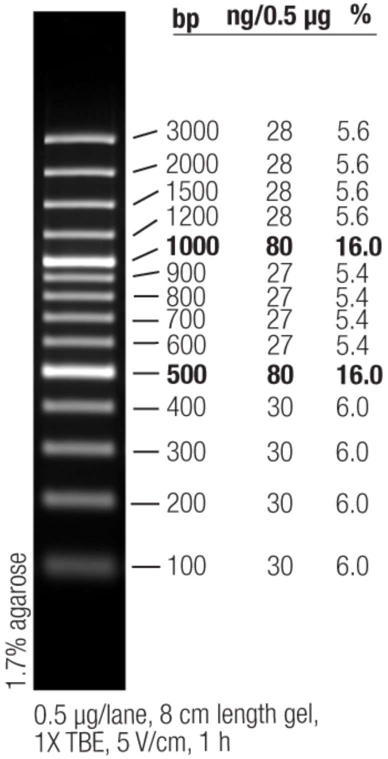

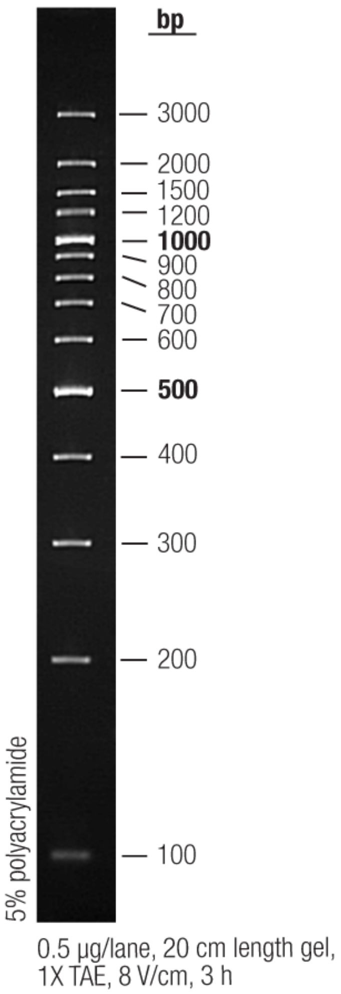

https://www.thermofisher.com/document-connect/document-connect.html?url=https://assets.thermofisher.com/TFS-Assets%2FLSG%2Fmanuals%2FMAN0013008_GeneRuler_100bp_Plus_DNALadder_50ug_UG.pdf

PageRuler™ Plus Prestained Protein Ladder, 10 to 250 kDa; Catalog number: 26620

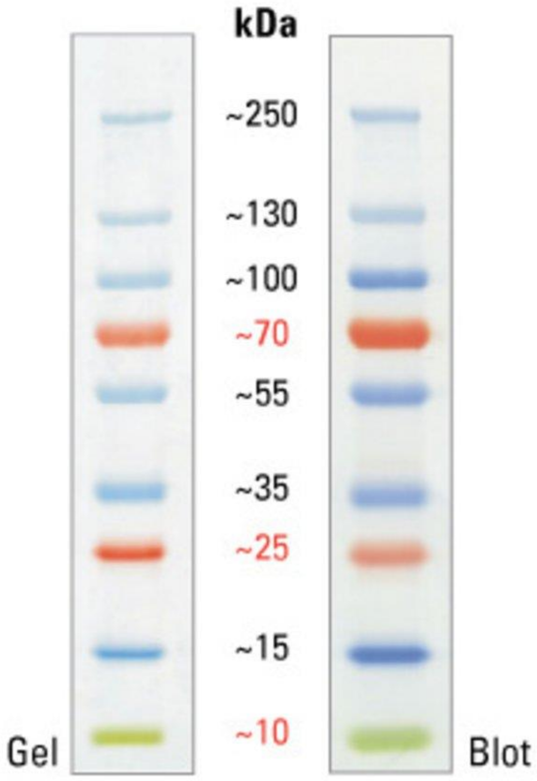

https://www.thermofisher.com/order/catalog/product/26620?SID=srch-srp-26620

Native agarose gels were captured on C-80 Epi-Illumination UV Darkroom.

SDS-PAGE and WB were scaned on HP psc 1315 al-in-one or HP Deskjet 3055A.

Fig_2_SDS-PAGE_mechanical inoculation-capRNA

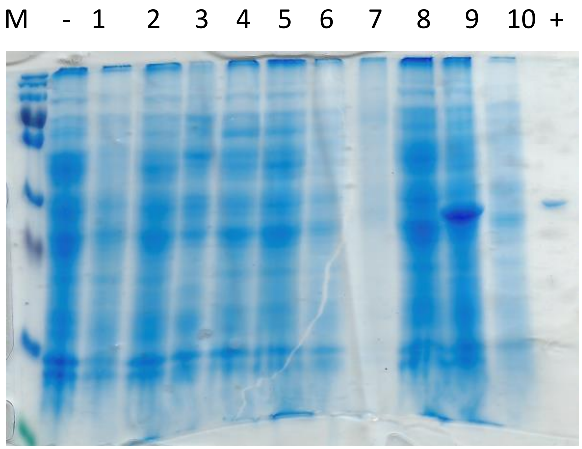

Fig_2_SDS-PAGE_Helios Gene Gun-capRNA

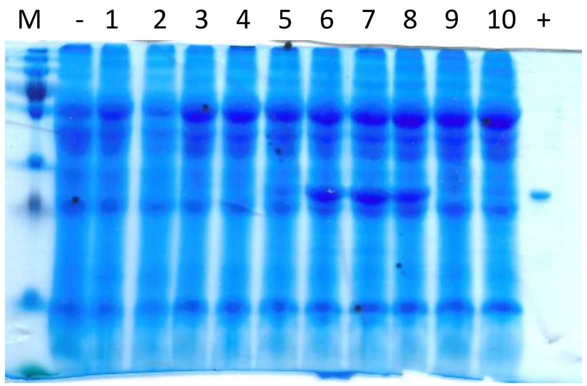

"-" – mock plants; 1-10 – inoculated oat plants with corresponding cDNA RNA transcript; "+" – purified WT RGMoV as positive control

Fig_2_SDS-PAGE_mechanical inoculation-RNA

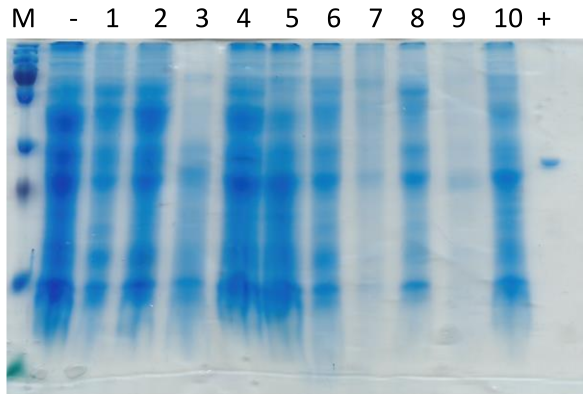

Fig_2_SDS-PAGE_mechanical inoculation-RGMoV

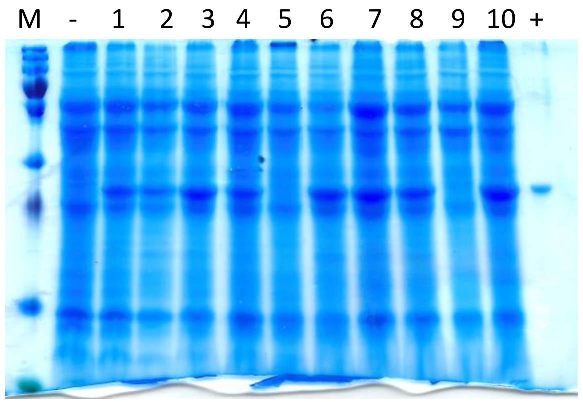

Fig_2_WB_mechanical inoculation-capRNA

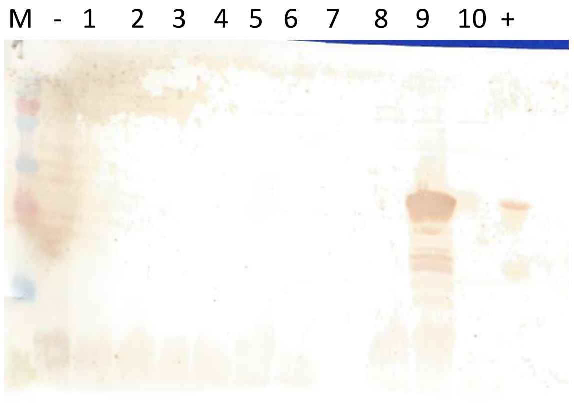

Fig_2_WB_Helios Gene Gun-capRNA

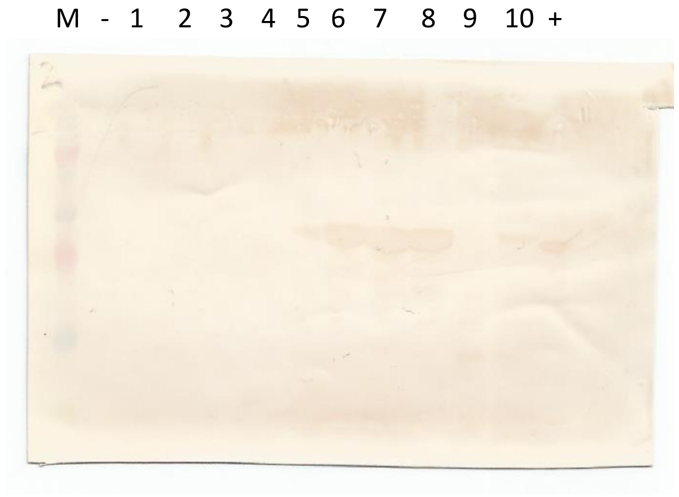

"-" – mock plants; 1-10 – inoculated oat plants with corresponding cDNA RNA transcript; "+" – purified WT RGMoV as positive control

Fig_2_WB_mechanical inoculation-RNA

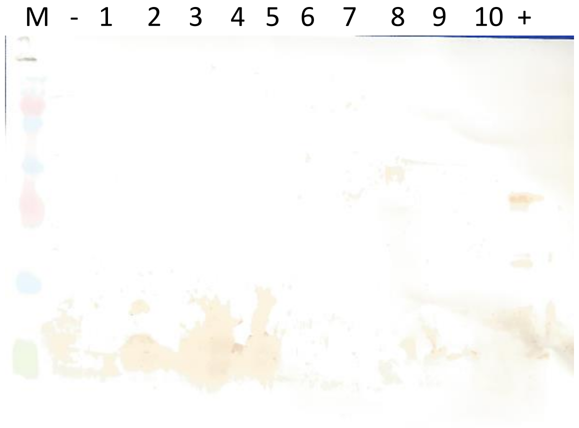

Fig_2_WB_mechanical inoculation-RGMoV

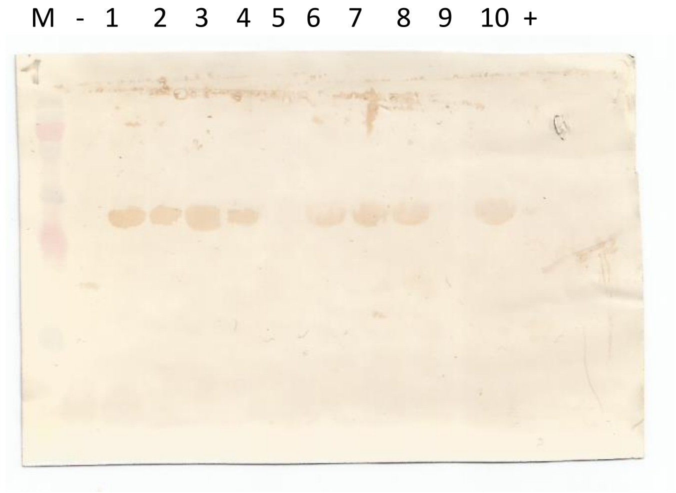

Fig_2_NAG_mechanical inoculation-capRNA_RNA

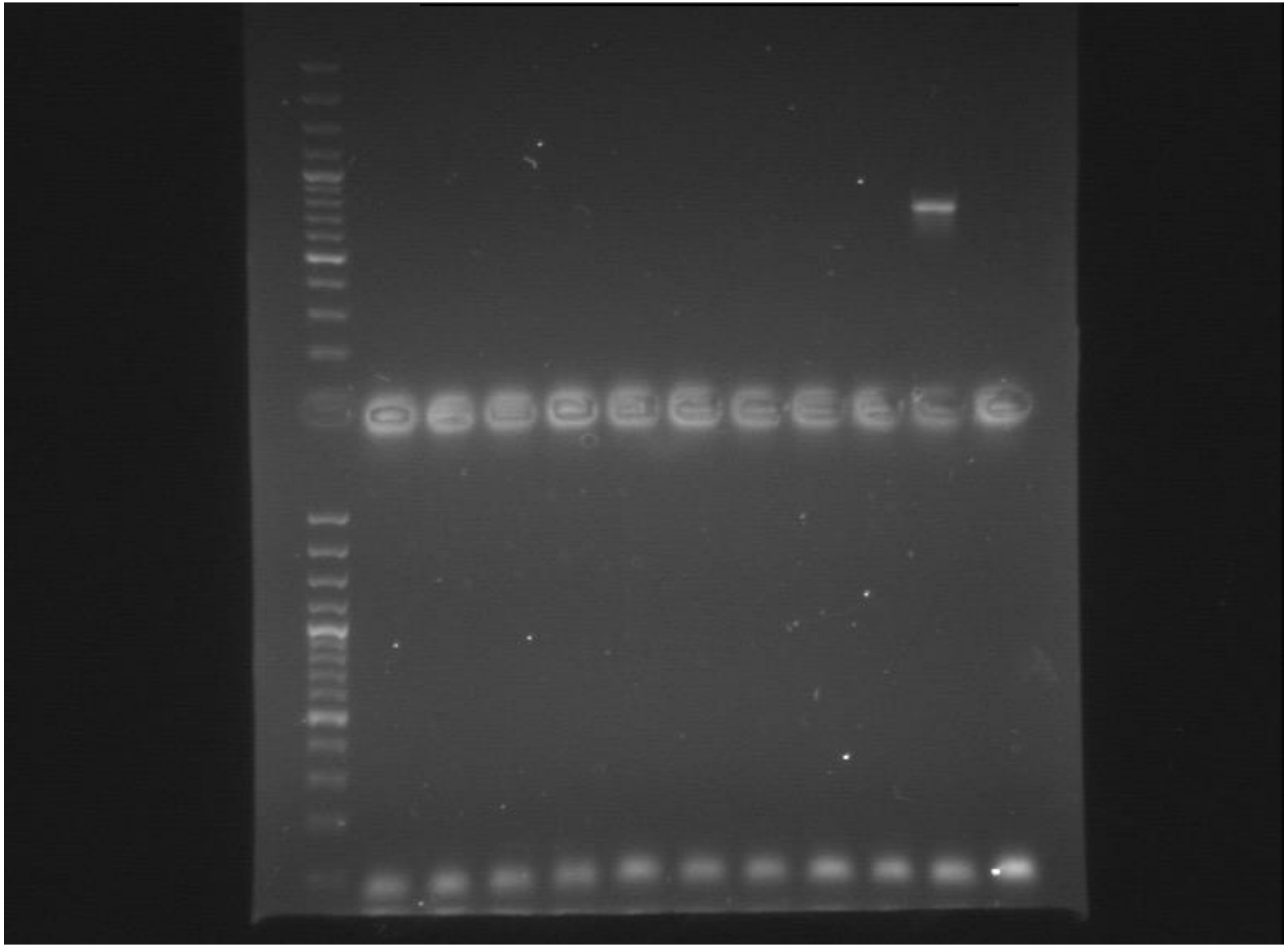

"-" – mock plants; 1-10 – inoculated oat plants with corresponding cDNA RNA transcript.

Fig_2_NAG_mechanical inoculation-RGMoV_Helios Gene Gun-capRNA

M - 1 2 3 4 5 6 7 8 9 10

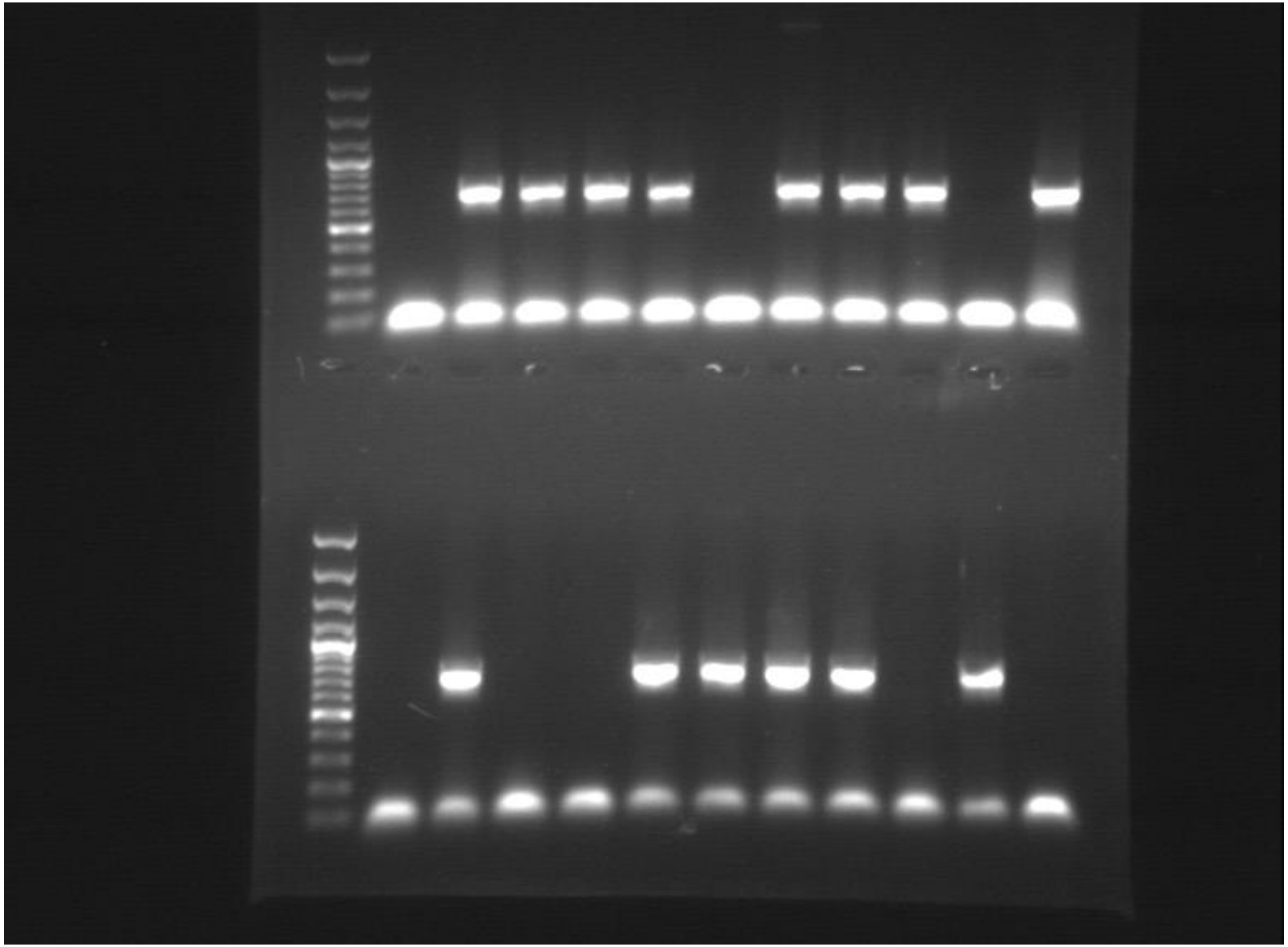

“-” – mock plants; 1-10 – inoculated oat plants with corresponding cDNA RNA transcript.

Fig_S1_SDS-PAGE_pJET-RGMoV-cDNAp

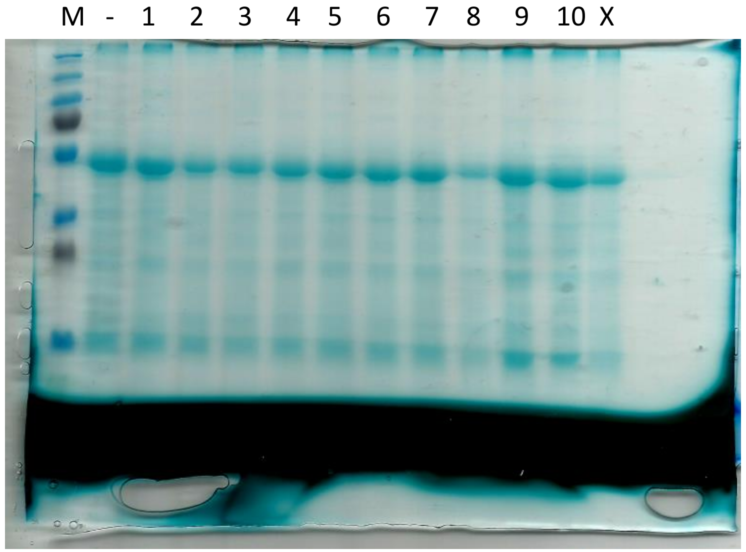

Fig_S1_SDS-PAGE_pJET-RGMoV-cDNAp-3end

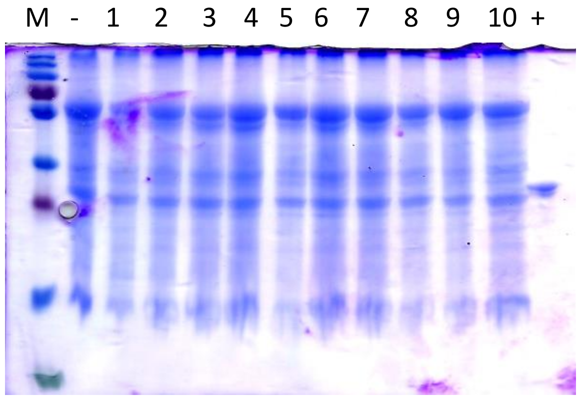

“-” – mock plants; 1-10 – inoculated oat plants with corresponding cDNA RNA transcript; “+” – purified WT RGMoV as positive control

Fig_S1_SDS-PAGE_pJET-RGMoV-cDNAp-5UTR-new-PGM

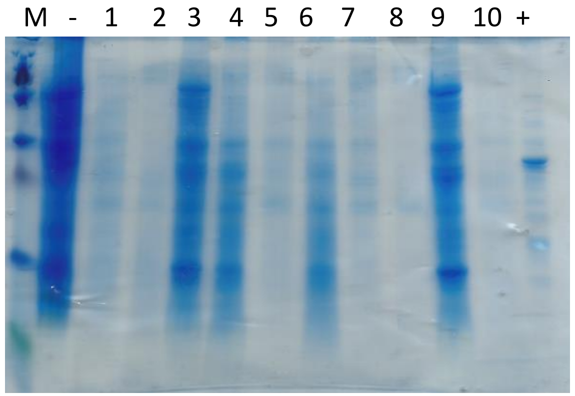

Fig_S1_SDS-PAGE_pJET-RGMoV-cDNAp-5UTR-new-short-PGM

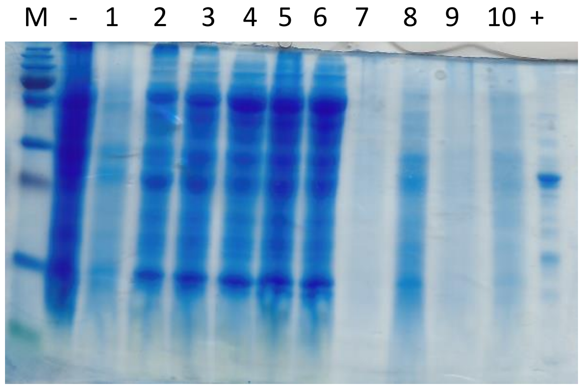

Fig_S1_SDS-PAGE_pTZ-T7-RGMoV-cDNA-3end-5UTR-new-PGM

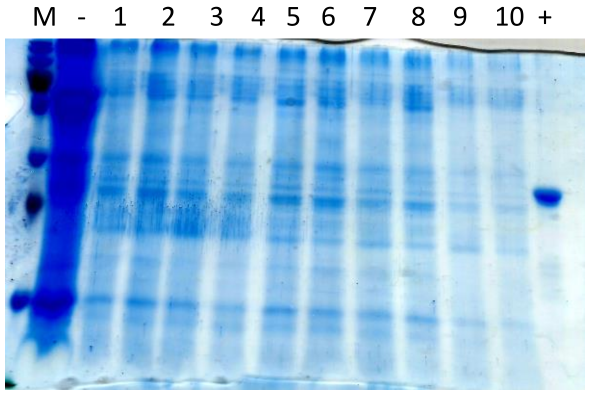

Fig_S1_WB_pJET-RGMoV-cDNAp

M - 1 2 3 4 5 6 7 8 9 10

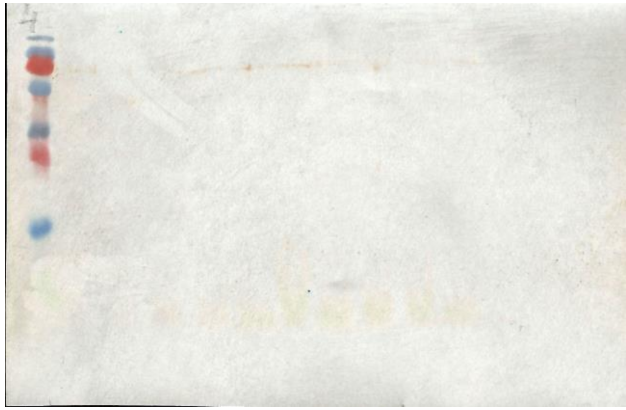

Fig_S1_WB_pJET-RGMoV-cDNAp-3end

M - 1 2 3 4 5 6 7 8 9 10 +

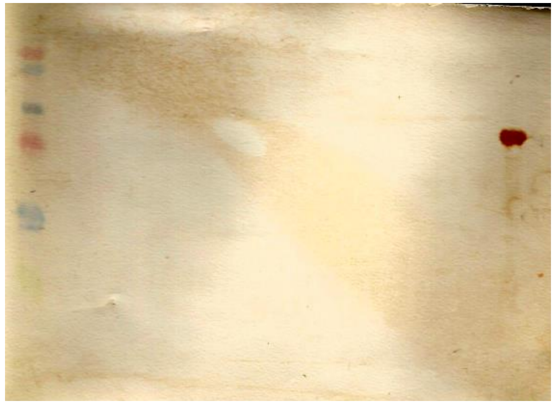

“-” – mock plants; 1-10 – inoculated oat plants with corresponding cDNA RNA transcript; “+” – purified WT RGMoV as positive control

Fig_S1_WB_pJET-RGMoV-cDNAp-5UTR-new-PGM

M - 1 2 3 4 5 6 7 8 9 10 +

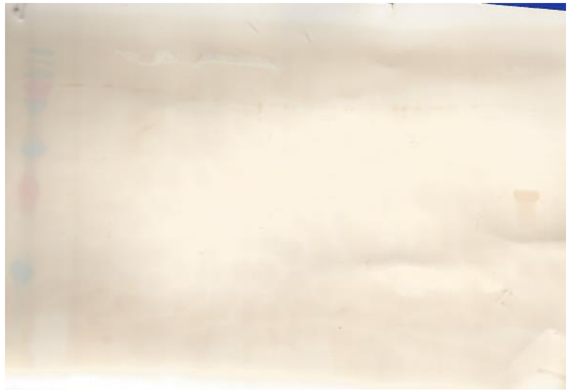

Fig_S1_WB_pJET-RGMoV-cDNAp-5UTR-new-short-PGM

M - 1 2 3 4 5 6 7 8 9 10 +

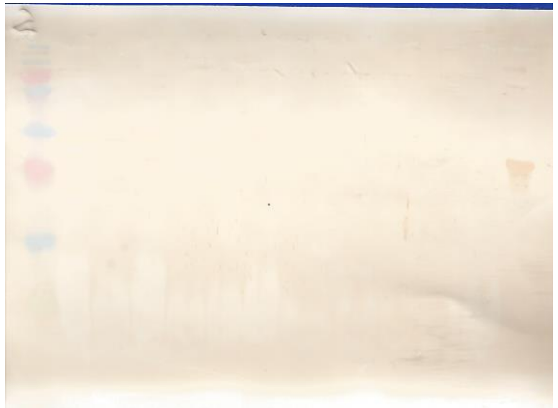

Fig_S1_WB_pTZ-T7-RGMoV-cDNA-3end-5UTR-new-PGM

M - 1 2 3 4 5 6 7 8 9 10 +

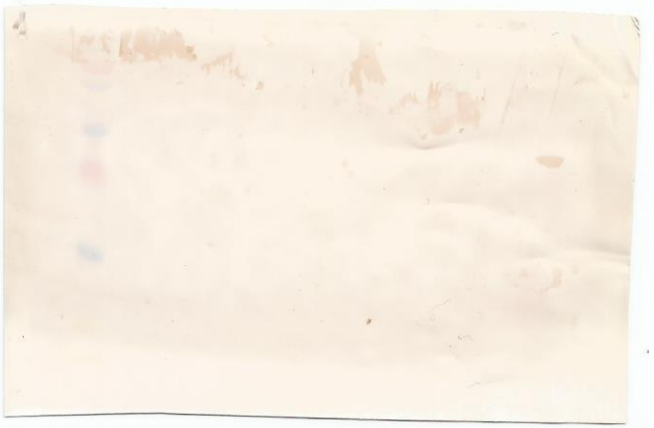

Supplement: S1 Raw images — Red arrows indicate possible RGMoV T = 1 particles; the white bar represents the 500 nm scale. (PDF) [file pone.0287278.s003.pdf]
